# Food Enzymes: Essential Factors of Complete Nutrition

**Published:** 1918-06

**Authors:** Thomas G. Read

**Affiliations:** D.M.D. Harvard, L.D.S.ENG.


### FOOD ENZYMES: ESSENTIAL FACTORS OF COMPLETE NUTRITION

BY THOMAS G. READ, D.M.D. HARVARD, L.D.S.ENG.

The effects of the actions of the enzymes were first studied by the brewing industry, which at the beginning was concerned in the enzyme diastase or amylase of malt, and then in the enzymes of yeast.

The pathology of beer was so unfolded that diseases to which it was liable are now practically eliminated.

The enzyme diastase or amylase was first observed in 1814 to be responsible for changing starch into sugar, and in 1831 it was discovered that saliva possessed a very similar property, which is due to the enzyme ptyalin contained in the saliva. It is a great pity the matter was not then further investigated, because it would have been found that conversions by enzymes contained in the human organism are not sufficient alone to maintain complete nutrition. When the enzyme amylase of starchy foodstuffs has been destroyed before acting, no conversion of starch into sugar takes place during cooking, and when this food reaches the mouth starch is converted by the enzyme ptyalin of the saliva into sugar. Lactic acid is rapidly formed from this, forming sugar by acid-producing bacteria, which are always present in the mouth. Ptyalin and acid-producing bacteria are swallowed with the bolus of starchy food, and the formation of lactic acid continues for some time. The lactic acid combines with alkaline bases. The neutral salts formed are excreted by the kidneys and skin, and the body deprived of essentials of complete nutrition.

Forming or nascent lactic acid has a great affinity to combine with lime salts. Thus, besides doing harm while passing through the system by combining with alkaline bases, there is also a great tendency for nascent lactic acid, while forming in the mouth, to combine with lime salts of the enamel of teeth and start dental decay.

Over fifty enzymes are now known to exist, of which the individuality and mode of action have been described. The French have the soundest teeth in Europe. The bread of France is still the Staff of Life, because it is made from flour containing the unimpaired enzymes of the grain.

Our war bread is most indigestible and harmful, because our enzymes of the grain are destroyed by millers while making our war flour. Thus when water of a temperature of about 80 degrees F. is added to the flour, no conversions by the enzymes amylase and cellulase of the germ take place to render starch and cellulose soluble, that they may be digested, and no action of the enzyme cerealin of the aleurone layer takes place, analogous to trypsin of pancreatic juice, by converting the protein of the grain into soluble peptones.

The change of our bread from the Staff of Life to the present degraded bread we are now supplied with dates from the introduction into this country, in 1862, of the roller-mill, which was invented that the wheat-germs containing the enzymes amylase and cellulase might be removed from the flour during milling. A drying plant has been added to the roller-mill. It is used to dry grain after washing while conditioning wheat. If too much heat is developed during drying, the enzymes amylase, cellulase and cerealin are destroyed. When the chemistry of food enzymes is as well unfolded as the chemistry of beer, there will be a great decrease in human suffering, and many prevalent diseases may disappear, particularly those described as deficiency diseases.—*The London Medical Times.*

